# Design and Uncertainty Evaluation of a Calibration Setup for Turbine Blades Vibration Measurement

**DOI:** 10.3390/s24248050

**Published:** 2024-12-17

**Authors:** Lorenzo Capponi, Giulio Tribbiani, Vittoria Medici, Sara Fabri, Andrea Prato, Paolo Castellini, Alessandro Schiavi, Nicola Paone, Gianluca Rossi

**Affiliations:** 1 Department of Industrial Engineering and Mathematical Sciences, Polytechnic University of Marche, Via Brecce Bianche 12, 60128 Ancona, Italy; v.medici@staff.univpm.it (V.M.);; 2 Department of Industrial Engineering, University of Perugia, Via G. Duranti 93, 06125 Perugia, Italy; giulio.tribbiani@phd.unipd.it (G.T.); sara.fabri@unipg.it (S.F.);; 3 INRIM—National Institute of Metrological Research, Strada delle Cacce, 91, 10135 Torino, Italy; a.prato@inrim.it (A.P.); a.schiavi@inrim.it (A.S.)

**Keywords:** uncertainty, tip timing, metrology, turbomachinery

## Abstract

Turbomachinery engines face significant failure risks due to the combination of thermal loads and high-amplitude vibrations in turbine and compressor blades. Accurate stress distribution measurements are critical for enhancing the performance and safety of these systems. Blade tip timing (BTT) has emerged as an advanced alternative to traditional measurement methods, capturing blade dynamics by detecting deviations in blade tip arrival times through sensors mounted on the stator casing. This research focuses on developing an analytical model to quantify the uncertainty budget involved in designing a calibration setup for BTT systems, ensuring targeted performance levels. Unlike existing approaches, the proposed model integrates both operational variability and sensor performance characteristics, providing a comprehensive framework for uncertainty quantification. The model incorporates various operating and measurement scenarios to create an accurate and reliable calibration tool for BTT systems. In the broader context, this advancement supports the use of BTT for qualification processes, ultimately extending the lifespan of turbomachinery through condition-based maintenance. This approach enhances performance validation and monitoring in power plants and aircraft engines, contributing to safer and more efficient operations.

## 1. Introduction

The complex combination of thermal loads with uncontrolled high-amplitude vibrations of turbine blades can induce turbomachinery engines to failure [1]. Measuring and monitoring the stress distribution on turbine engines components in operation is the key for accurately informing numerical models, either predictive or diagnostic, which can reduce fatigue damage, and to increase the performance and safety of the machinery [2]. Strain-gauges have been historically used as a reference for measuring the dynamics of rotating blades, because of their high accuracy and well-established signal processing algorithms [3,4]. However, they have reduced lifetimes in high-temperature conditions, are intrusive, and provide information limited to the instrumented blades, in addition to requiring complex means of transmitting data from a rotating system, and long machinery downtime [5]. Several alternatives to strain-gauge-based monitoring have been proposed in the past 50 years, leveraging vibration-, temperature-, and ultrasound-based techniques [6]. Nowadays, the blade tip timing (BTT) approach is one of the most advanced and versatile in situ techniques for axial turbomachinery blade dynamics measurements [7,8]. The tip timing technique derives stress distributions in rotating blades from blade tip vibration amplitudes estimated by measuring delays or advances in the arrival time of the blade tip at fixed angular positions using sensors radially installed on the casing: if the blade does not deflect, its time-of-arrival (ToA) in front of the sensors is defined by the geometries of the setup and of the machinery (and its dynamics); if a blade deflects at specific rotating conditions (i.e., at or close to resonant frequencies), a delay or advance in the ToA, with respect to the rigid body, of the blade in front of the sensors is expected and measured [9,10]. Modern BTT measurement systems (BTTMSs), used by leading manufacturing industries for power generation and aircraft engines, employ optical fibers with total-reflection sensors, rather than eddy current, microwave, magnetoresistive, or capacitance sensors, because of their dynamical performances and reliability in critical and harsh environments [11,12,13,14].

In the last twenty years, great effort has been dedicated to BTT system characterization and development. For instance, in the 1990s, Andrenelli et al. [15,16] and Nava et al. [17] presented designs and characterizations of non-intrusive BTT setups, comparing those results to the ones obtained with strain-gauge measurements. More recently, Zhu et al. [18] proposed an accelerated algorithm to improve accuracy and efficiency in identifying vibration parameters from under-sampled signals. Gao et al. [19] proposed a fitting methodology to improve blade tip timing accuracy by reducing errors from speed fluctuations, validated through simulations and experiments. Finally, Zhao et al. [20] developed an iterative signal space algorithm to improve the accuracy of blade vibration parameter identification from under-sampled BTT signals, demonstrating its effectiveness and robustness through simulations and experiments. A large part of this effort has been devoted to studying the measurement uncertainty of tip timing systems. In fact, the assessment of procedures to estimate the measurement uncertainty and to ensure traceability represents an enabling factor for effective uptake and application of BTTMS for turbomachinery certification, as well as for accurate monitoring and diagnostics [21]. Rossi et al. [22] proposed an arrival-time uncertainty model for optical probes used in BTT systems focusing on rise time effects; Russhard et al. [23] outlined qualitative weights of potential uncertainty sources in BTT measurements, and Pan et al. [24] have broadened previous studies to multi-modal conditions. Zhou et al. [25] proposed a numerical-based analysis for identifying vibration parameters and estimating BTT uncertainty caused by rotational speed fluctuation. More recently, Mohamed et al. [26] presented a process for validating the finite element stress and deflection predictions of aero-engine compressor blades under non-rotation conditions, giving the quantified uncertainties associated with numerical modeling and the measurement processes. Capponi et al. [27] experimentally investigated arrival time uncertainty due to measurement parameters and data processing of a typical commercial measurement system. Similarly, Tocci et al. [28] proposed a sensitivity analysis of data-processing parameters for determining deflection amplitude from arrival time information in commercial software. Tribbiani et al. proposed a comparative model of the uncertainty on arrival time measured with and without a reference sensor [29]. Finally, data modeling and uncertainty analysis was recently investigated via machine learning and Bayesian methods by Liu et al. [30] and Wang et al. [31], respectively. Nevertheless, the literature still lacks a complete analytical model that gives the uncertainty on the blade deflection amplitude, accounting for the many uncertainty sources, taking into account their combination according to the guidelines for uncertainty in measurement [32]. The objective of this metrological effort is to address this challenge and bridge this gap.

This research seeks to establish the groundwork for a larger framework within which the authors intend to develop a procedure and an experimental setup for calibrating tip timing measurement systems. As a first contribution to this, the goal of this study is to build a flexible and comprehensive analytical model for determining the uncertainty on the blade tip vibration amplitude to be used to inform the design of a BTT calibration setup. To achieve this, a physical model for determining the form of the blade tip deflection amplitudes from the arrival time samples and the machinery characteristics is elaborated. From this model, the derivation of the standard uncertainty on the tip deflection amplitude is obtained, where the analytical form of the main sources of uncertainty is explored. The result of this work enables us to determine the uncertainty on the blade deflection during the design of a BTT calibration setup. In this way, operational conditions and machinery parameters can be adjusted to achieve lower (and known) uncertainty values.

## 2. Fundamentals of Tip Timing

### 2.1. Blade Deflection from Measured Arrival Time

In a simplified schematic of a turbomachinery dynamics, the blade tip law of motion p(t) can be seen as the composition of two contributions, defined in Equation (1) as the circular motion around the rotor axis (first term) and the tip vibration around the underformed condition s(t):

(1) $$p\left(t\right)=R\;\int_{0}^{t}\omega \left(\tau \right)\;d\tau +s\left(t\right),$$

where ω(t) represents the time modulation of the rotational speed ω in the time interval [0, *t*], and *R* is the tip radius, sum of the rotor radius $R_{r}$ and blade length $R_{b}$. If the blades behave as rigid bodies (i.e., no deflection occurs), Equation (1) simplifies to the first term. In this case, the arrival time of the *i*-th blade in front of a sensor radially installed on the casing corresponds to the $\Delta t_{i,k}^{rot}$, which can be defined at the *k*-th revolution with

(2) $$\Delta t_{i,k}^{rot}=\frac{\theta }{\omega },$$

where θ is the angle between two consecutive probes, and ω is the perfectly constant rotational speed of the shaft. However, in real operating conditions, where the rotational speed is not constant, the rotational speed $\bar{\omega }$ averaged on *M* revolutions needs to be considered, and Equation (2) becomes

(3) $$\Delta t_{i,k}^{rot}=\frac{\theta }{\bar{\omega }}.$$

**Figure 1:**
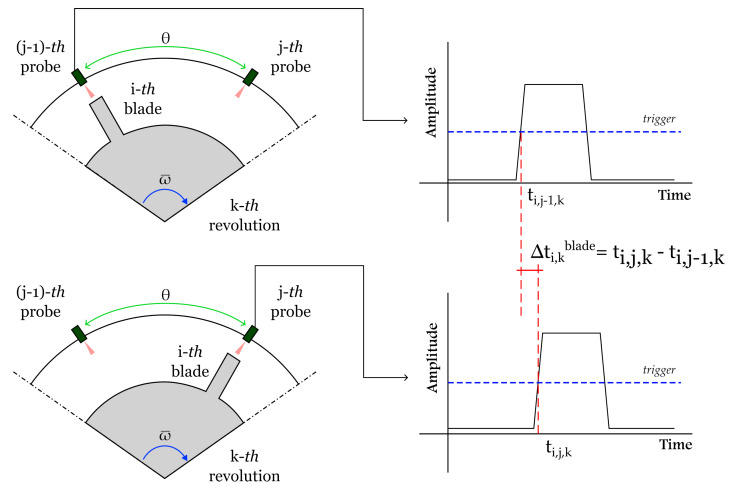
Measurement of arrival times of blade tips.

On the other hand, when the blades behave as flexible bodies and their deflections are considered, the actual arrival time $\Delta t_{i,k}^{blade}$ is

(4) $$\Delta t_{i,k}^{blade}=\Delta t_{i,k}^{rot}+\Delta t_{i,k}^{vib},$$

where $\Delta t_{i,k}^{vib}$ is the delay (or advance) in arrival time measured by the BTT technique. With regard to Equation (1), the *i*-th blade deflection amplitude due to vibration can be now defined at the *k*-th revolution as follows:

(5) $$s_{i,k}^{vib}=v_{t}\;\Delta t_{i,k}^{vib},$$

where $v_{t}$ is the tangential speed of the blade tip:

(6) $$v_{t}=\bar{\omega }\;R,$$

and $\Delta t_{i,k}^{vib}$ can be calculated using

(7) $$\Delta t_{i,k}^{vib}=\Delta t_{i,k}^{blade}-\Delta t_{i,k}^{rot}=\left(t_{i,j,k}-t_{i,j-1,k}\right)-\frac{\theta }{\bar{\omega }},$$

where $t_{i,j,k}$ and $t_{i,j-1,k}$ are the time instants of transit of the same blade in front of the *j*-th sensor and in front of the previous one (i.e., (j−1)-th sensor), respectively, at the *k*-th revolution. Figure 1 represents the way the time samples $t_{i,j,k}$ and $t_{i,j-1,k}$ are taken in a standard BTT system.

Substituting Equations (6) and (7) into Equation (5), for a given pair of BTT probes (*j* and j−1), we obtain

(8) $$s_{i,k}^{vib}=\bar{\omega }\;R\;\left[\left(t_{i,j,k}-t_{i,j-1,k}\right)-\frac{\theta }{\bar{\omega }}\right]=R\;\left[\bar{\omega }\;\left(t_{i,j,k}-t_{i,j-1,k}\right)-\theta \right].$$

Equation (8) allows us to determine the amplitude of the blade tip vibration $s_{i,k}^{vib}$ if the average rotational speed $\bar{\omega }$, the tip radius *R*, the angle θ, and the time samples $t_{i,j,k}$ are known. Moreover, Equation (8) highlights the four potential sources of uncertainty on the vibration displacement (i.e., $\bar{\omega }$, *R*, θ, and $t_{i,j,k}$). The effect of these sources will be investigated throughout this research.

In the broader context of turbomachinery blade dynamics analysis, blade deflection $s_{i,k}^{vib}$ serves as the foundational input for determining the stress/strain states of the blade. Accurately quantification of the uncertainty in these data ensures reliable validation of potential fatigue damage and enhances the predictive accuracy of numerical models. This, in turn, supports iterative improvements in the design and reliability of components, ultimately leading to safer and more efficient turbomachinery systems.

### 2.2. Methods for Measuring the Average Rotational Speed

In this section, different ways to evaluate the average rotational speed $\bar{\omega }$ of the rotor are analyzed. The idea behind this step is to untie the average rotational speed evaluation from the tip timing measurement, enabling a simplified form of the uncertainty on $s_{i,k}^{vib}$.

From the fundamental laws of motion, the average rotational speed $\bar{\omega }$ of a rotating body (e.g., a rotor) can be seen as the ratio of a covered angle β and the average time interval $\Delta t_{\beta }^{rot}$ taken to sweep β:

(9) $$\bar{\omega }=\frac{\beta }{\Delta t_{\beta }^{rot}}.$$

**Figure 2:**
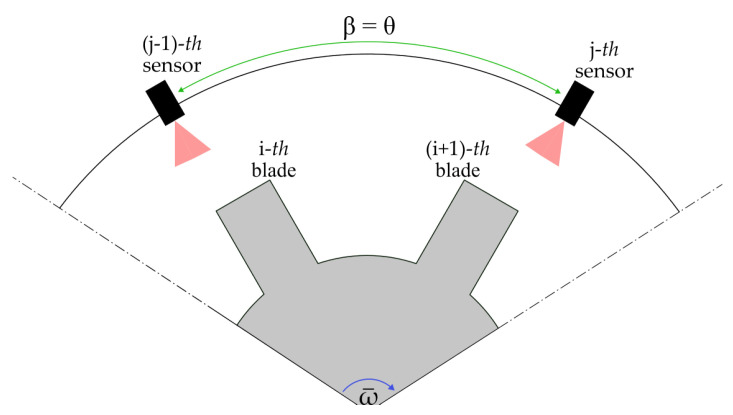
Scenario where β assumes the value of the angle between two consecutive sensors θ.

Depending on how the time interval $\Delta t_{\beta }^{rot}$ is measured, different angles need to be considered. If $\Delta t_{\beta }^{rot}$ is the time needed by a single blade to sweep between two consecutive sensors, then β is θ (i.e., the angle between two sensors), and $\Delta t_{\theta }^{rot}$ can be expressed as

(10) $$\Delta t_{\theta }^{rot}=\frac{\sum_{z=1}^{M}\left(t_{i,j,z}-t_{i,j-1,z}\right)}{M},$$

where $t_{i,j,z}$ and $t_{i,j-1,z}$ (with z=1…M) are the time samples used for determining the rotational speed averaged on *M* revolutions. With regard to this, the authors mean that the rotational speed is evaluated as a first step (on *M* revolutions), while the blade tip times of arrival are investigated in a separate instance (on *N* revolutions). This measuring solution is presented in Figure 2.

Moreover, while summing time instants, if any subscript assumes a null value (e.g., j−1 with j=1), the time instant is picked as reference and its value is zero as well. In this way, Equation (8) becomes

(11) $$\begin{array}{cc} s_{i,k}^{vib} & =R\;\left[\bar{\omega }\;\left(t_{i,j,k}-t_{i,j-1,k}\right)-\theta \right]\\ \; & =R\;\left[\frac{\theta }{\frac{\sum_{z=1}^{M}\left(t_{i,j,z}-t_{i,j-1,z}\right)}{M}}\;\left(t_{i,j,k}-t_{i,j-1,k}\right)-\theta \right]\\ \; & =\theta \;R\;\left[\frac{M\;(t_{i,j,k}-t_{i,j-1,k})}{\sum_{z=1}^{M}\left(t_{i,j,z}-t_{i,j-1,z}\right)}-1\right]. \end{array}$$

**Figure 3:**
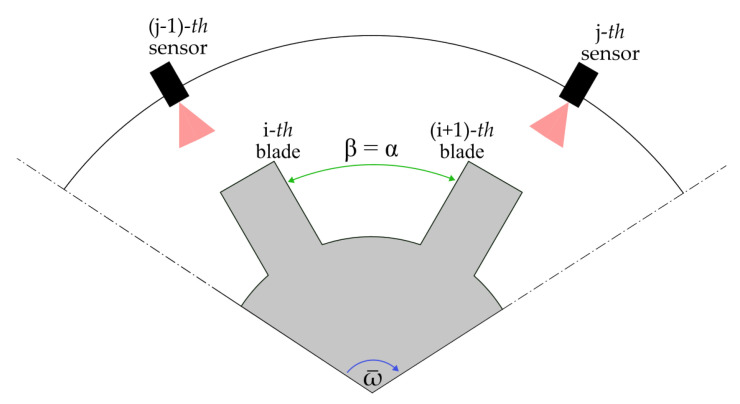
Scenario where β assumes the value of the angle between two blades α.

Similarly, if the time interval $\Delta t_{\beta }^{rot}$ is the time needed by two consecutive blades to transit in front of a single sensor, then β is the spacing angle α between two blades (see Figure 3), and $\Delta t_{\alpha }^{rot}$ is

(12) $$\Delta t_{\alpha }^{rot}=\frac{\sum_{z=1}^{M}\left(t_{j,z}-t_{j,z-1}\right)}{M},$$

where *M* is the number of time events (which does not correspond to the number of revolutions).

In this case, Equation (8) becomes

(13) $$\begin{array}{cc} s_{i,k}^{vib} & =R\;\left[\bar{\omega }\;\left(t_{i,j,k}-t_{i,j-1,k}\right)-\theta \right]\\ \; & =R\;\left[\frac{\alpha \;M\;(t_{i,j,k}-t_{i,j-1,k})}{\sum_{z=1}^{M}\left(t_{j,z}-t_{j,z-1}\right)}-\theta \right]. \end{array}$$

Otherwise, when external systems are used to determine $\bar{\omega }$ (e.g., once-per-rev sensors, phonic wheel, or zebra tape), $\Delta t_{\beta }^{rot}$ is taken as the time passed between two notches or stripes, β is the spacing angle σ between the external sensor references, and $\Delta t_{\sigma }^{rot}$ is

(14) $$\Delta t_{\sigma }^{rot}=\frac{\sum_{z=1}^{M}\left(t_{z}^{ref}-t_{z-1}^{ref}\right)}{M},$$

where $t_{z}^{ref}$ are the time samples corresponding to the reference events used to calculate $\bar{\omega }$. In this case, Equation (8) becomes

(15) $$\begin{array}{cc} s_{i,k}^{vib} & =R\;\left[\bar{\omega }\;\left(t_{i,j,k}-t_{i,j-1,k}\right)-\theta \right]\\ \; & =R\;\left[\frac{\sigma \;M\;(t_{i,j,k}-t_{i,j-1,k})}{\sum_{z=1}^{M}\;\left(t_{z}^{ref}-t_{z-1}^{ref}\right)}-\theta \right]. \end{array}$$

**Figure 4:**
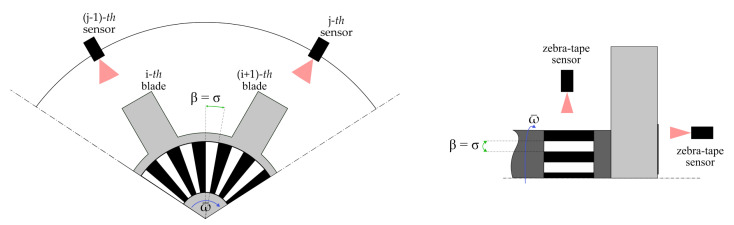
Scenario where β is the spacing angle σ between the external sensor references.

This measuring scenario is shown in Figure 4, where a zebra-tape-based system is used as an example.

In this context, σ would be the angle between tape stripes, which could be mounted either on the front face of the bladed rotor or on its shaft. This solution is used in Section 5 as a test case for the design of a potential BTT calibration setup.

## 3. Uncertainty Model for Blade Tip Deflection

In Section 2.2, the analytical form of the blade tip deflection due to blade vibration was proposed for three different scenarios, where the rotational speed is measured using one blade sweeping in front of two sensors (Equation (11)), two blades in front of the same sensor (Equation (13)), or using a dedicated measurement system (Equation (15)). Regardless of the specific case, the uncertainty on the blade tip vibration amplitude can be seen as a function of the angle chosen for measuring the rotational speed, the radius, and the time samples as follows:

(16) $$s_{i,k}^{vib}=s_{i,k}^{vib}\left(\beta ,\theta ,R,t_{k},t_{z}\right),$$

The uncertainty model for the blade tip deflection can be derived using the methodologies defined in the Guide to the Expression of Uncertainty in Measurement (GUM) [32], so the propagated uncertainty, computed while taking into account variances $u^{2}\left(s_{i,k}^{vib}\right)$ on $s_{i,k}^{vib}$ from Equation (16), can be written as follows:

(17) $$\begin{array}{cc} u^{2}\left(s_{i,k}^{vib}\right) & ={\left(\frac{\partial s}{\partial \beta }\right)}^{2}\;u^{2}\left(\beta \right)+{\left(\frac{\partial s}{\partial \theta }\right)}^{2}\;u^{2}\left(\theta \right)+{\left(\frac{\partial s}{\partial R}\right)}^{2}\;u^{2}\left(R\right)\\ \; & +{\left(\frac{\partial s}{\partial t_{k}}\right)}^{2}\;u^{2}\left(t_{k}\right)+{\left(\frac{\partial s}{\partial t_{z}}\right)}^{2}\;u^{2}\left(t_{z}\right). \end{array}$$

Equation (17) can also be seen as follows:

(18) $$\begin{array}{cc} u^{2}\left(s_{i,k}^{vib}\right) & =c_{u(\beta )}\;u^{2}\left(\beta \right)+c_{u(\theta )}\;u^{2}\left(\theta \right)+c_{u(R)}\;u^{2}\left(R\right)\\ & +c_{u(t_{k})}\;u^{2}\left(t_{k}\right)+c_{u(t_{z})}\;u^{2}\left(t_{z}\right), \end{array}$$

where $c_{u}$ are the squared partial derivatives of the single uncertainty contributions.

In the case where β assumes the value of the angle between two consecutive sensors θ, Equation (17) can be expanded using Equation (11):

(19) $$\begin{array}{cc} u^{2}\left(s_{i,k}^{vib}\right) & ={\left(R\;\left[\frac{M\;(t_{i,j,k}-t_{i,j-1,k})}{\sum_{z=1}^{M}\left(t_{i,j,z}-t_{i,j-1,z}\right)}-1\right]\right)}^{2}u^{2}\left(\theta \right)\\ \; & +{\left(\theta \left[\frac{M\;(t_{i,j,k}-t_{i,j-1,k})}{\sum_{z=1}^{M}\left(t_{i,j,z}-t_{i,j-1,z}\right)}-1\right]\right)}^{2}u^{2}\left(R\right)\\ \; & +2{\left(\frac{\theta \;R\;M}{\sum_{z=1}^{M}\left(t_{i,j,z}-t_{i,j-1,z}\right)}\right)}^{2}u^{2}\left(t_{k}\right)\\ \; & +2\;M{\left(\frac{\theta \;R\;M\;(t_{i,j,k}-t_{i,j-1,k})}{{\left(\sum_{z=1}^{M}\left(t_{i,j,z}-t_{i,j-1,z}\right)\right)}^{2}}\right)}^{2}u^{2}\left(t_{z}\right) \end{array}$$

The coefficients 2 and 2M on $u^{2}\left(t_{k}\right)$ and $u^{2}\left(t_{z}\right)$, respectively, arise from the assumption that, for uncorrelated time events, $u^{2}\left(t_{i,j,k}\right)=u^{2}\left(t_{i,j-1,k}\right)$ (here, the coefficient “2”), and $u^{2}\left(t_{i,j,z}\right)=u^{2}\left(t_{i,j-1,z}\right)$ is valid over the sum in *M* instances (here, the coefficient “2*M*”). This hypotheses is valid for all the following scenarios.

Similarly, in the case where β assumes the value of the angle between two consecutive blades α, and considering that $u^{2}\left(t_{i,j,k}\right)=u^{2}\left(t_{i,j-1,k}\right)$ and $u^{2}\left(t_{j,z}\right)=u^{2}\left(t_{j,z-1}\right)$, Equation (17) can be seen using Equation (13) as follows:

(20) $$\begin{array}{cc} u^{2}\left(s_{i,k}^{vib}\right) & =R^{2}u^{2}\left(\theta \right)\\ \; & +{\left(\frac{\alpha \;M\;(t_{i,j,z}-t_{i,j-1,z})}{\sum_{z=1}^{M}\left(t_{j,z}-t_{j,z-1}\right)}-\theta \right)}^{2}u^{2}\left(R\right)\\ \; & +2{\left(\frac{R\;\alpha \;M}{\sum_{z=1}^{M}\left(t_{j,z}-t_{j,z-1}\right)}\right)}^{2}u^{2}\left(t_{k}\right)\\ \; & +2\;M{\left(\frac{R\;\alpha \;M\;(t_{i,j,z}-t_{i,j-1,z})}{{\left(\sum_{z=1}^{M}\left(t_{j,z}-t_{j,z-1}\right)\right)}^{2}}\right)}^{2}u^{2}\left(t_{z}\right)\\ \; & +{\left(\frac{R\;M\;(t_{i,j,z}-t_{i,j-1,z})}{\sum_{z=1}^{M}\left(t_{j,z}-t_{j,z-1}\right)}\right)}^{2}u^{2}\left(\alpha \right) \end{array}$$

Analogously, in the case where β assumes the value of the angle between two reference stripes or notches σ, and considering that $u^{2}\left(t_{i,j,k}\right)=u^{2}\left(t_{i,j-1,k}\right)$ and $u^{2}\left(t_{z}^{ref}\right)=u^{2}\left(t_{z-1}^{ref}\right)$, when using Equation (15), Equation (17) becomes

(21) $$\begin{array}{cc} u^{2}\left(s_{i,k}^{vib}\right) & =R^{2}u^{2}\left(\theta \right)\\ \; & +{\left(\frac{\sigma \;M\;(t_{i,j,z}-t_{i,j-1,z})}{\sum_{z=1}^{M}\left(t_{z}^{ref}-t_{z-1}^{ref}\right)}-\theta \right)}^{2}u^{2}\left(R\right)\\ \; & +2{\left(\frac{R\;\sigma \;M}{\sum_{z=1}^{M}\left(t_{z}^{ref}-t_{z-1}^{ref}\right)}\right)}^{2}u^{2}\left(t_{k}\right)\\ \; & +2\;M{\left(\frac{R\;\;\sigma \;M\;(t_{i,j,z}-t_{i,j-1,z})}{{\left(\sum_{z=1}^{M}\left(t_{z}^{ref}-t_{z-1}^{ref}\right)\right)}^{2}}\right)}^{2}u^{2}\left(t_{z}^{ref}\right)\\ \; & +{\left(\frac{R\;M\;(t_{i,j,z}-t_{i,j-1,z})}{\sum_{z=1}^{M}\left(t_{z}^{ref}-t_{z-1}^{ref}\right)}\right)}^{2}u^{2}\left(\sigma \right) \end{array}$$

The authors acknowledge that consecutive time measurements $t_{j}$ and $t_{j-1}$ were taken using the same measurement device, which can potentially introduce correlation between these measurements. Although the uncertainty values were initially assumed equal ($u\left(t_{j}\right)=u\left(t_{j-1}\right))$, this does not inherently imply that the correlation coefficient between these measurements is zero. In fact, the covariance between time measurements may propagate into the overall uncertainty. If this correlation is significant, it can affect the accuracy of the combined uncertainty estimation. In this study, while initial analyses suggested that correlation contribution might be negligible, we recognize that this assumption should be justified. Future studies will incorporate a qualitative assessment of covariance effects or, where applicable, numerical evaluations of correlation coefficients between repeated measurements.

## 4. Sources of Uncertainty in Tip Timing

### 4.1. Uncertainty on Tip Radius

The uncertainty u(R) mainly depends on the blade deformation, on the blade manufacturing tolerance, and on the measurement uncertainty of the instrumentation used to assess the tip radius *R* (type-B uncertainty) [32]. To obtain a low uncertainty, instruments with high accuracy and precision need to be employed.

For larger blade deflections and high dynamical regimes, two effects would need to be accounted for when determining the uncertainty on the tip radius. The first one is linked to the axial shortening of the blade due to its deflection, caused by both the aerodynamic load and the vibration contribution. In this case, the blade length $R_{b}$ would actually be $R-\Delta R_{b}$. However, this effect occurs for much larger deflections than those of BTT systems, and $-\Delta R_{b}$ can be considered negligible.

An opposite effect would happen due to the centrifugal force: in this case, the blade would experience an elongation of about $+\Delta R_{b}$. Similarly, this effect is negligible with respect to the uncertainty in the measurement of the tip radius *R*.

### 4.2. Uncertainty on Angle

The uncertainty in the measurement of a generic angle β (i.e., θ, α, σ), denoted as u(β), depends primarily on the resolution of the instrument and other factors, such as stability and repeatability under the specific conditions of measurement [32]. In this work, the uncertainty u(β) is considered a type A uncertainty [32]. For this purpose, measurement uncertainties lower than $1^{\prime \prime }$ can be achieved using high-precision instrumentation, such as clinometers or laser goniometers [33].

### 4.3. Uncertainty on Time Sample

The uncertainty u(t) of a generic measurement of a time sample *t* (i.e., $t_{i,j,k}$, $t_{i,j-1,k}$, $t_{i,j,z}$, $t_{i,j,z-1}$, $t_{z}^{ref}$, $t_{z-1}^{ref}$) is given by two different contributions in the form of variance as follows:

(22) $$u^{2}\left(t\right)=u^{2}\left(t_{1}\right)+u^{2}\left(t_{2}\right).$$

The first contribution $u^{2}\left(t_{1}\right)$ depends on the dynamical performances of the sensor used. Particularly, its bandwidth $f_{3dB}$ would be ideally as wide as possible, to allow the measurement of blade passages producing a pulse having a high-slope rising edge. For this reason, the rise time of the signal is investigated in detail. The rise time of the signal $t_{1}$ is the combination of three contributions: the characteristic time of the sensor $t_{1s}$, which describes the ability of the sensor to acquire high-speed transient phenomena; the time $t_{1p}$, which depends on the rising-edge slope of the signal; the characteristic time $t_{1o}$, which is proper of the acquisition system (and generally negligible with respect to the other contributions) [22]:

(23) $$t_{1}=\sqrt{t_{1s}^{2}+t_{1p}^{2}+t_{1o}^{2}}.$$

In this step, optical sensors are considered, as they are among the most frequently employed due to their higher metrological properties. In optical sensors, photo-detectors are used to convert the light intensity reflected by the blades, and captured by the fiber optics, into electrical signals. The behavior of RC-circuit-based photo-detectors can be modeled as a first-order response. While other BTT probes (e.g., magneto-resistive sensors) might be described by more complex models, the rise time $t_{1s}$ for optical sensors is defined as

(24) $$t_{1s}=\frac{0.35}{f_{3dB}}.$$

On the other hand, the rise time $t_{1p}$ can be seen as the ratio of the diameter of the sensitive spot *d* of the sensor and the tangential speed $v_{t}$ of the blade [22]:

(25) $$t_{1p}=\frac{d}{v_{t}}.$$

In this way, Equation (23) becomes

(26) $$t_{1}=\sqrt{{\left(\frac{0.35}{f_{3dB}}\right)}^{2}+{\left(\frac{d}{v_{t}}\right)}^{2}}.$$

**Figure 5:**
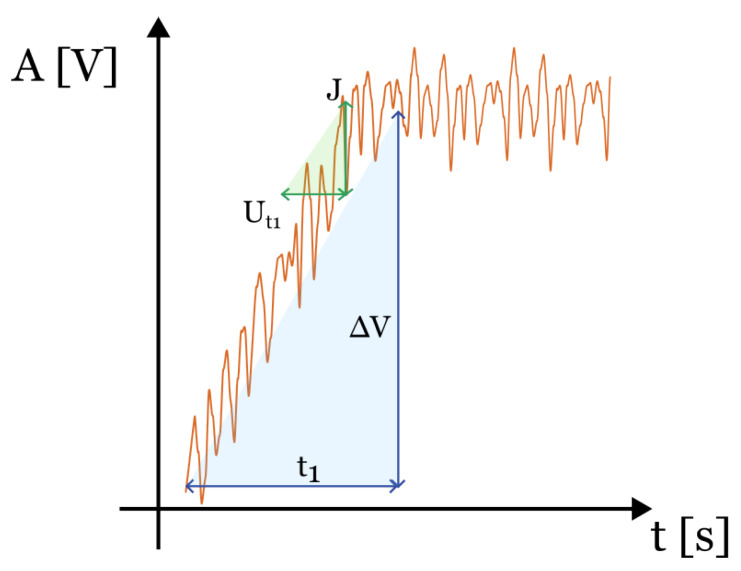
Rise time $t_{1}$ of a signal of amplitude ΔV: similarity of triangle built with the variability interval of the time samples $U_{t_{1}}$ and the signal noise *J* [22].

The uncertainty $u(t_{1})$ on $t_{1}$ can be obtained from the similarity of triangles shown in Figure 5, where the time $t_{1}$ and the amplitude of the pulsed signal ΔV are compared to the variability interval (of a uniform distribution) $U_{t_{1}}$ and to the noise *J* as follows:

(27) $$\frac{J}{U_{t_{1}}}=\frac{\Delta V}{t_{1}}.$$

With $\frac{\Delta V}{J}$ being the signal-to-noise ratio ϵ, $U_{t_{1}}$ becomes

(28) $$U_{t_{1}}=\frac{1}{\epsilon }\sqrt{{\left(\frac{0.35}{f_{3dB}}\right)}^{2}+{\left(\frac{d}{v_{t}}\right)}^{2}}$$

Considering $U_{t_{1}}/2$ as the half-amplitude of a uniform distribution, the variance of the uncertainty $u^{2}\left(t_{1}\right)$ on $t_{1}$ can be defined as follows:

(29) $$u^{2}\left(t_{1}\right)=\frac{{\left(\frac{U_{t_{1}}}{2}\right)}^{2}}{3}=\frac{{\left(\frac{1}{2\;\epsilon }\sqrt{{\left(\frac{0.35}{f_{3dB}}\right)}^{2}+{\left(\frac{d}{v_{t}}\right)}^{2}}\right)}^{2}}{3}=\frac{{\left(\frac{1}{\epsilon }\sqrt{{\left(\frac{0.35}{f_{3dB}}\right)}^{2}+{\left(\frac{d}{v_{t}}\right)}^{2}}\right)}^{2}}{12}.$$

Similarly, the term $u^{2}\left(t_{2}\right)$ in Equation (22) is given by the resolution uncertainty of the acquisition system, which can be fairly approximated with the inverse of the sampling frequency $f_{c}$. In this case, the probability distribution is hypothesized to be of rectangular shape, where its half-amplitude is $f_{c}/2$:

(30) $$u^{2}\left(t_{2}\right)=\frac{{\left(\frac{1}{2}\cdot \frac{1}{f_{c}}\right)}^{2}}{3}=\frac{{\left(\frac{1}{f_{c}}\right)}^{2}}{12}.$$

Finally, the uncertainty in the time sample $u^{2}\left(t\right)$ can be written from Equations (29) and (30) in its variance form as follows:

(31) $$u^{2}\left(t\right)=\frac{{\left(\frac{1}{\epsilon }\sqrt{{\left(\frac{0.35}{f_{3dB}}\right)}^{2}+{\left(\frac{d}{v_{t}}\right)}^{2}}\right)}^{2}+{\left(\frac{1}{f_{c}}\right)}^{2}}{12}.$$

## 5. Design of a Calibration Setup for Tip Timing Measurements

The primary focus of this work is the design of a calibration setup for blade tip timing (BTT) measurement systems, leveraging the uncertainty models developed in previous sections. This design aims to ensure the accurate evaluation of blade deflection under targeted conditions defined by the turbomachinery’s geometry, dynamics, and operational parameters. Specifically, we consider a scenario where an external measurement system, such as an optical sensor monitoring a zebra tape affixed to the shaft, is used to determine the average rotation speed. The uncertainty model outlined in Equation (21) serves as the foundation for constructing the uncertainty budget related to the BTT measurements. Table 1 presents typical values for a potential calibration setup, reflecting realistic conditions and constraints for accurate blade deflection assessments.

The resulting variables and the main uncertainties on the values in Table 1 are evaluated by means of the built physical and uncertainty models, and given in Table 2. Notably, the uncertainties u(σ) and u(θ) share the same value, as both angles are supposed to be measured with the same instrument, ensuring consistency in their uncertainty contributions.

Finally, the coefficients $c_{u}$ of the uncertainty terms in Equation (18) are computed so that the weight of the single uncertainty contributions on the overall uncertainty on the blade deflection amplitude sample can be determined, which are reported with a 95% confidence level in Table 3.

Two main conclusions can be drawn from the results in Table 2 and Table 3. The uncertainties on the dimensional features have the largest impact on the overall uncertainty. This means that the measurements of blade radius *R* and angles σ and θ require high accuracy and precision by means of high-end instrumentation and solid procedures. On the other end, uncertainty in time samples have the lowest weight on the blade deflection amplitude samples. In fact, as expected, instruments for measuring time events are nowadays characterized by high performances, which do not limit BTT measurement uncertainty.

## 6. Conclusions

To avoid failures of turbomachinery components, an accurate experimental characterization of their structural behavior unveils hidden criticalities which can be addressed and solved in the machinery design phase. As an alternative to strain-gauge-based techniques, blade tip timing was proposed in the 1970s for measuring blade tip deflection amplitudes in situ and in operating conditions. The information of the measurement uncertainty on the blade tip deflection is the key for properly informing numerical models and validating turbomachinery operation.

This research aimed to lay the first stone of a larger framework, within which the authors intend to build a calibration procedure and setup for tip timing measurement systems. Specifically, a physical model, produced to describe the blade tip law of motion in simplified turbomachinery dynamics, was used to build an uncertainty model on the blade tip deflection in different operating scenarios. For this reason, sources of uncertainty were identified and given an analytical description. Finally, these models were used to design a potential BTT calibration experimental setup: typical values of BTT measurements were used as targets, and the weights of the single uncertainty contributions on the overall uncertainty on the blade deflection amplitude samples were determined. As expected, the uncertainty in the dimensional features (i.e., tip radius and angles) were found to have the largest impact on the blade tip deflection, while the uncertainty in the time samples is almost negligible compared to them.

The forthcoming effort of this study will include the application of the developed models for the realization of a BTT calibration setup, where the uncertainties will be verified experimentally. Moreover, the influence of other designing and operational parameters will be investigated to bolster the uncertainty models presented herein.

## Figures and Tables

**Table 1:** Parameters of design.

Variable	Value	Unit
*R*	1.50 × $10^{1}$	m
*M*	1.00 × $10^{4}$	-
ω	2.00 × $10^{3}$	rad/s
$\Delta t^{blade}$	5.25 × $10^{-4}$	s
θ	1.05 × $10^{0}$	rad
σ	6.28 × $10^{-2}$	rad
ϵ	1.00 × $10^{2}$	-
$f_{3dB}$	1.00 × $10^{9}$	Hz
*d*	1.00 × $10^{-4}$	m
$v_{t}$	3.00 × $10^{2}$	m/s
$f_{c}$	1.00 × $10^{9}$	Hz
*s*	3.00 × $10^{-4}$	m

**Table 2:** Derived variables and uncertainties.

Variable	Value	Unit
u(R)	1.50 × $10^{-5}$	m
u(σ) & u(θ)	6.28 × $10^{-6}$	rad
u(t)	1.00 × $10^{-9}$	s

**Table 3:** Weights of the uncertainty contributions and overall uncertainty on the blade deflection amplitude sample, calculated with a 95% confidence level.

Variable	Value	Units
$c_{u(\theta )}\cdot u^{2}\left(\theta \right)$	8.88 × $10^{-13}$	$m^{2}$
$c_{u(R)}\cdot u^{2}\left(R\right)$	2.47 × $10^{-10}$	$m^{2}$
$c_{u(t_{k})}\cdot u^{2}\left(t_{k}\right)$	5.00 × $10^{-20}$	$m^{2}$
$c_{u(t_{z}^{ref})}\cdot u^{2}\left(t_{z}^{ref}\right)$	5.07 × $10^{-15}$	$m^{2}$
$c_{u(\sigma )}\cdot u^{2}\left(\sigma \right)$	2.48 × $10^{-10}$	$m^{2}$
u(s)	4.45 × $10^{-5}$	m
u(s)/s	14.8	%

## Data Availability

Data are available upon request.

## Nomenclature

The following nomenclature is used in this manuscript:

*^ref^*	Superscript for average rotational speed measurement system
$c_{u(q)}$	Coefficient of uncertainty on the *q* variable
*d*	Sensor spot diameter (m)
$f_{c}$	Sampling frequency of measurement system (Hz)
*J*	Signal noise (V)
*k*	Index for rotor revolution for determining deflection amplitude
*M*	Number of time events for determining average rotational speed
*N*	Number of revolutions for determining deflection amplitude
p(t)	Blade tip law of motion as sum of rotation and vibration (m)
*q*	Generic variable
*R*	Radius (m)
$R_{b}$	Blade length (m)
$R_{r}$	Rotor radius (m)
*s*	Deflection sample (m)
s(t)	Blade tip law of motion due to vibration (m)
*t*	Time sample (s)
$t_{1}$	Rise time (s)
$t_{2}$	Acquisition time resolution (s)
$t_{1o}$	Characteristic time of the acquisition system (s)
$t_{1p}$	Characteristic time due to the rising-edge slope of the signal (s)
$t_{1s}$	Characteristic time of the sensor (s)
$U_{t_{1}}$	Variability interval of time samples (uniform distribution) (s)
$v_{t}$	Blade tangential speed (m/s)
*z*	Index for rotor time events for determining averaged rotational speed
α	Angle between two consecutive blades (rad)
β	Generic angle (rad)
$\Delta R_{b}$	Variation of blade length (m)
$\Delta t^{blade}$	Blade tip arrival time (s)
$\Delta t^{rot}$	Blade tip arrival time due to rotation (s)
$\Delta t^{vib}$	Blade tip arrival time due to vibration (s)
ΔV	Pulsed signal amplitude (V)
ϵ	Signal-to-noise ratio (dB)
ω	Rotational speed (rad/s)
$\bar{\omega }$	Rotational speed averaged on *M* revolutions (rad/s)
σ	Angle between two consecutive references for measuring average rotational speed (rad)
θ	Angle between two consecutive BTT probes (rad)
$f_{3dB}$	Bandwidth of the sensor (Hz)
u(q)	Uncertainty on the *q* variable
